# How Outreach Training and Supportive Supervision (OTSS) Affect Health Facility Readiness and Health-Care Worker Competency to Prevent and Treat Malaria in Niger: A Secondary Analysis of OTSS Data

**DOI:** 10.4269/ajtmh.23-0359

**Published:** 2024-02-06

**Authors:** Daniel Koko, Djibrilla Arouna, Yves-Marie Bernard, Thierno Ba, Jadmin Mostel, Yahaya Abdou, Eric Coulibaly, Zilahatou Bahari-Tohon, Lawrence M. Barat

**Affiliations:** ^1^ PMI Impact Malaria Project, Population Services International, Niamey, Republic of Niger;; ^2^ PMI Impact Malaria Project, Population Services International, Washington, District of Columbia;; ^3^ National Malaria Program, Niamey, Republic of Niger;; ^4^ U.S. President’s Malaria Initiative, U.S. Agency for International Development, Niamey, Republic of Niger

## Abstract

The quality of health services is key to the goal of averting morbidity and mortality from malaria. From July 2020 to August 2021, PMI Impact Malaria supported the implementation of four rounds of Outreach Training and Supportive Supervision (OTSS) in 12 health districts in the two regions of Niger: Dosso and Tahoua. Through OTSS, trained supervisors conducted onsite visits to observe an average of 174 healthcare workers (HCWs) per round in 96 public primary health facilities, managing persons with fever or conducting antenatal care (ANC) consultations, and then provided instant and individualized feedback and onsite training. Data from health facility readiness, case management, and malaria in pregnancy (MiP) checklists across the four rounds were analyzed using Wilcoxon’s and the χ^2^ tests. These analyses highlighted improved facility readiness, including an increased likelihood that HCWs had received classroom training, and facilities had increased availability of guidelines and algorithms by round 4 compared with round 1. Median HCW performance scores showed an improvement in the correct performance and interpretation of malaria rapid diagnostic tests, in classification of malaria as uncomplicated or severe, and in the management of uncomplicated malaria across the four rounds. For MiP services, malaria prevention and the management of pregnant women with malaria also improved from round 1 to round 4. These findings provide further evidence that OTSS can achieve rapid improvements in health facility readiness and HCW competency in managing outpatients and ANC clients.

## INTRODUCTION

Malaria is the major cause of morbidity and mortality in sub-Saharan Africa. According to the WHO, in 2021 the African region was the most affected, with 95% and 96% of global cases and deaths, respectively, and with two thirds of total deaths from malaria occurring among children younger than 5 years old.^1^ Key to averting morbidity and mortality from malaria and other febrile illnesses is the skill of health-care workers (HCWs) to make an accurate and timely diagnosis based on history, physical examination, and diagnostic testing to guide proper treatment. Systematic reviews have identified modest effects of stand-alone training and supportive supervision^2^^,^^3^ on the management of malaria and other febrile illnesses, with potential stronger effects when both training and supervision are used together.^4^^,^^5^

In one of largest systematic reviews of strategies to improve health worker performance in low- and middle-income countries, Rowe et al.^6^ demonstrated that training or supervision alone produced moderate effects in terms of appropriate treatment. However, when these interventions were combined, their impact on service provision was greater. Strategies that combined training, supervision, and various forms of community support generated the greatest impact on provider performance. Training and supervision form the core of many countries’ quality improvement (QI) efforts, although these activities are not usually paired in a systematic way in most countries.

In Niger, the National Malaria Program (NMP) has built the capacity of health workers to manage uncomplicated malaria cases and malaria in pregnancy (MiP) primarily through classroom training; however, challenges remain in bridging the gap between national malaria guidelines and clinical practice.^7^ Niger was selected as a U.S. President’s Malaria Initiative (PMI) partner country in fiscal year 2017. Since 2019, PMI Impact Malaria has supported the NMP to strengthen capacity in malaria case management and MiP through the implementation of a QI approach in primary health facilities (PHFs) in the two target regions of Dosso and Tahoua.

**Figure 1.**
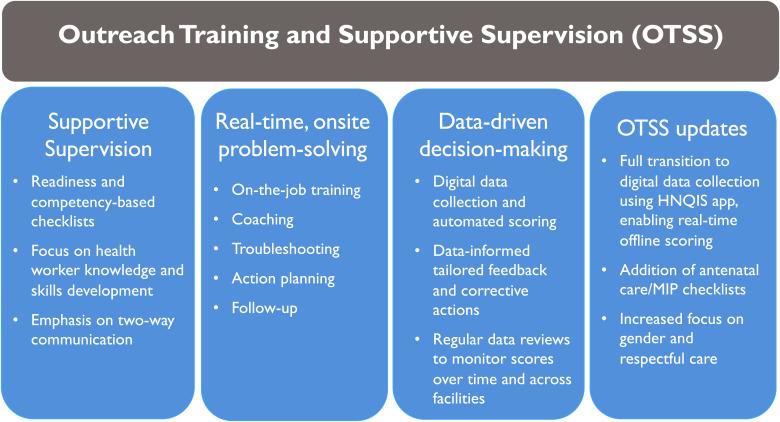
OTSS components. Improvements from previous OTSS efforts are discussed on page 4 of this supplement. HNQIS = Health Network Quality Improvement System; OTSS = Outreach Training and Supportive Supervision.

The cornerstone for QI is the Outreach Training and Supportive Supervision (OTSS) approach (Figure 1). Using the OTSS approach, trained supervisors conduct onsite visits to health facilities using a tablet-based standardized checklist on the Health Network Quality Improvement System (HNQIS) app to observe and interview directly clinical staff conducting a consultation with a person with fever or performing an antenatal care (ANC) consultation, and to assess facility readiness, such as the availability of commodities, equipment, and case management and MiP guidelines and algorithms. Supervisors provided in real time, individualized feedback and onsite training to HCWs based on the steps in the consultation that deviated from national guidelines. They also developed action plans together with facility staff to address broader health facility issues. OTSS data were also used to identify needs for additional classroom training, and provision of guidelines, algorithms, commodities, and equipment needed for quality malaria service provision. A more detailed description of the OTSS approach can be found in Barat et al.^8^ in this supplement.

Supervision data are uploaded and compiled automatically in the PMI Impact Malaria data hub, which uses District Health Information System 2 software (Version 2.36.10, University of Oslo, Oslo, Norway).

Using the OTSS data set maintained in the PMI Impact Malaria data hub, we evaluated the effects of OTSS on public health facility readiness and health worker performance in meeting clinical and prevention standards for malaria case management in outpatient and antenatal consultations.

## MATERIALS AND METHODS

### Program setting and population.

From July 2020 to August 2021, 12 health districts were supported by the PMI Impact Malaria Niger Project to improve malaria case management through implementation of clinical and MiP OTSS in the Dosso and Tahoua regions. Selection of these two regions was a joint programmatic decision of the NMP and the PMI regarding where to target PMI support for multiple malaria interventions, which was based primarily on the high malaria burden in these regions. Almost all PHFs in these 12 areas received OTSS visits. Four quarterly rounds of OTSS were undertaken from July 2020 through August 2021, assessing an average of 174 HCWs in approximately 96 PHFs (99 PHFs in April 2021 and 93 in August 2021). A round is defined as a specific period during which a targeted set of facilities receives an OTSS visit. Each round of supervision targeted the same facilities, although a few facilities were not visited because of security concerns or competing activities at those facilities during the OTSS round.

Within health facilities, all staff who performed clinical consultations for the management of febrile illness and ANC were observed by district supervisors using the case management and MiP checklists, respectively, and facility readiness was assessed using a standard checklist.

### Study design.

This study involved a secondary analysis of OTSS data generated by the facility readiness checklist, outpatient department (OPD) checklist, and the MiP checklist (Supplemental Figure 1A–C, respectively). To estimate the effect of OTSS on facility readiness and on HCW performance by individual visit and over time, the individual steps in the checklists were weighted and scored to determine provider competency or facility readiness at the aggregate level (with the maximum competency being 100%) and to obtain a facility score. Our study assessed the effects of OTSS on variables listed in Table 1, including facility readiness factors, competency in the management of sick children in the OPD, and competency in the prevention of MiP and the management of malaria in pregnant women at the ANC clinic. Details of the components of each indicator are available in Supplemental Table 1.

**Table 1:** Description of variables

Variables	Checklist	Definition	Type
The availability of trained staff	Facility readiness	All categories having received classroom training in the past 2 years in malaria case management	Binary
The availability the malaria rapid diagnostic test	Facility readiness	The presence of mRDTs at the facility today (supervision day)	Binary
The availability at least one formulation of ACT	Facility readiness	The presence of at least one formulation of ACT at the facility (supervision day)	Binary
The availability of case management and MiP guidelines/algorithms	Facility readiness	The presence of case management and MiP guidelines/algorithms at the facility (supervision day)	Continuous
HCW competency in using and reading mRDTs	mRDT observation	Percentage score on the checklist for assessing the correct steps for performing and reading the mRDT	Continuous
HCW adherence to negative mRDT results	OPD	Percentage of HCWs who complied with the negative test results	Continuous
HCW competency of classifying malaria cases as uncomplicated or severe	OPD	Competency of HCWs in classifying malaria cases	Continuous
HCW competency in managing patients with uncomplicated malaria	OPD	Percentage score in history taking, clinical examination, and treating uncomplicated malaria cases with an ACT according to national guidelines	Continuous
HCW competency in the prevention of MiP	MiP	Percentage score in the provision of malaria preventive services during antenatal consultations, including administering intermittent preventive treatment during pregnancy, disseminating insecticide-treated bed nets, and providing correct counseling	Continuous
HCW competency in managing pregnant women with malaria	MiP	Percentage score in history taking, clinical examination, diagnosis, and treatment of cases according to national guidelines	Continuous

ACT = artemisinin-based combination therapy; HCW = health-care worker; MiP = malaria in pregnancy; mRDT = malaria rapid diagnostic test; OPD = outpatient department.

### Data management and analysis.

For each round of OTSS, data were entered in the HNQIS app, loaded onto tablet computers, and then compiled in the PMI Impact Malaria data hub. The study team extracted data from the data hub into Stata for data cleaning and analysis for the health facility readiness, case management, and MiP indicators. All data cleaning and statistical regressions were done using Stata/SE (version 15.1; Stata Corp, College Station, TX).

The structure of the data analysis consisted of selecting data elements of interest (Table 1) and assessing the performance of these variables in OTSS round 1 (R1) or round 2 (R2) versus round 4 (R4). To assess differences between scores from R1 or R2 and those from R4, we performed the Wilcoxon rank-sum test for continuous variables and the χ^2^ test for categorical variables. Specifically, variables such as using and reading malaria rapid diagnostic tests (mRDTs), adhering to a negative mRDT result, classifying malaria severity, managing uncomplicated malaria, preventing MiP, treating pregnant women with malaria, and the availability of mRDTs and case management and MiP guidelines were evaluated as continuous variables that were not normally distributed using the Wilcoxon rank-sum test. Categorical variables, such as the availability of trained staff, mRDTs, and at least one artemisinin-based combination therapy (ACT) formulation, were analyzed using the χ^2^ test. All statistical results are presented with a significance level set at *P* <0.05.

## RESULTS

The same PHFs were supported across 12 health districts in the regions of Dosso and Tahoua (Supplemental Figure 2). On average, 174 HCWs providing clinical consultations or ANC consultations in the selected PHFs were assessed by district supervisors. From July 2020 to August 2021, the four OTSS checklists were used to assess the readiness of the facilities visited (96 in R1 and 93 in R4), the use and reading of mRDTs in approximately 349 patients (113 in R1 and 109 in R4), the quality of malaria case management in 405 patients (99 in R1 and 101 in R4), and the management and prevention of MiP in 364 women (93 in R1 and 84 in R4).

The percentages of facilities scoring 90% or greater on the readiness and competency variables being assessed in our study are presented in Table 2. The proportion of PHFs with at least 50% of HCWs having received classroom training increased significantly from 28% in R2 to 48% in R4, 1 year later (*P* = 0.003, χ^2^ test) (Table 3). In the supervised PHFs, mRDT availability was high in both R1 and R4, at 99% and 94%, respectively (*P* = 0.09, χ^2^ test) (Table 3). The presence of at least one ACT formulation also was consistent across rounds: 98% in R1 and 96% in R4 (*P* = 0.430, χ^2^ test) (Table 3). Primary health facility availability of case management and MiP guidelines and algorithms increased significantly from R1 to R4, from 14% to 84% (*P* <0.001, Wilcoxon rank-sum test) (Table 3).

**Table 2:** Changes in readiness and competency scores of ≥90% from round 1 to round 4 in the Dosso and Tahoua regions, Niger, from July 2020 to August 2021

Variable	Round 1, *n*/*N *(%)	Round 2, *n*/*N *(%)	Round 3, *n*/*N *(%)	Round 4, *n*/*N *(%)
Availability of trained staff	–*	26/94 (28)	33/99 (33)	45/93 (48)
Availability of mRDTs	95/96 (99)	93/94 (99)	93/99 (94)	87/93 (94)
Availability of at least one formulation of ACT	94/96 (98)	92/94 (98)	95/99 (96)	89/93 (96)
Availability of case management and MiP guidelines/algorithms	13/96 (14)	66/94 (71)	85/99 (86)	78/93 (84)
HCW competency in using and reading mRDTs	53/113 (47)	86/106 (81)	18/21 (88)	87/109 (80)
HCW adherence to negative mRDT results	95/99 (96)	98/109 (90)	96/96 (10%)	101/101 (100)
HCW competency of classifying malaria cases as uncomplicated or severe	50/99 (50)	95/109 (87)	89/96 (93)	93/101 (92)
HCW competency in the management of patients with uncomplicated malaria	11/99 (11)	19/109 (17)	48/96 (50)	42/101 (42)
HCW competency in the prevention of MiP	25/93 (27)	51/100 (51)	63/87 (72)	68/84 (81)
HCW competency in managing pregnant women with malaria	21/93 (22)	66/100 (66)	72/87 (83)	71/84 (85)

ACT = artemisinin-based combination therapy; HCW = health-care worker; MiP = malaria in pregnancy; mRDT = malaria rapid diagnostic test.

* Training data were not available for round 1.

**Table 3:** Chi-square test results for categorical readiness variables

Variable	Baseline score (%)*	Endline score (%)	χ^2^ Value	*P*-value
Availability of trained staff^†^	28	48	8.5	0.03
Availability of at least one formulation of ACT	98	96	0.8	0.316
Availability of mRDTs^†^	99	94	4.0	0.09

ACT = artemisinin-based combination therapy; mRDT = malaria rapid diagnostic test.

* Reference value.

^†^ Baseline for mRDT availability and trained staff is round 2 because these data were not collected during round 1.

Assessing key competencies, improvements were observed from R1 to subsequent rounds (Table S1). In R1, only 47% of HCWs adhered correctly to guidelines on mRDTs performance and interpretation, which increased significantly to 80% by R4 (*P* <0.001, Wilcoxon rank-sum test) (Table 4). In contrast, HCWs adhered to the negative mRDT protocol during all rounds, with 96% in R1 and 100% in R4.

**Table 4:** Results of the Wilcoxon rank-sum test comparing median competency scores across continuous variables for the Dosso and Tahoua regions, Niger, from July 2020 to August 2021

Variable	Round 1 (%)	Round 4 (%)	z-Value	*P*-value
HCW competency in using and reading mRDTs	86	95	8.2	<0.001
HCW competency of classifying malaria cases as uncomplicated or severe	88	100	9.9	<0.001
HCW competency in the management of patients with uncomplicated malaria	37	75	−8.6	<0.001
HCW competency in the prevention of MiP	81	100	−2.1	0.034
HCW competency in managing pregnant women with malaria	85	100	−8.2	<0.001
Availability of case management and MiP guidelines/algorithms	83	100	−7.3	<0.001

HCW = health-care worker; MiP = malaria in pregnancy; mRDT = malaria rapid diagnostic test.

In R1, HCWs classified 50% of malaria cases correctly as either severe or uncomplicated. By R4, this value increased to 92% (Table 2). Competency in managing uncomplicated malaria cases also rose, from 11% in R1 to 42% in R4. The Wilcoxon rank-sum test verified these increases as statistically significant for both malaria classification and uncomplicated malaria management (*P* <0.001) (Table 4).

The proportion of ANC workers demonstrating competency in malaria prevention during pregnancy increased from 27% in R1 to 81% in R4 (*P* <0.001) (Table 4). In addition, ANC worker competency in managing pregnant women with malaria rose from 22% in R1 to 85% in R4 (*P* <0.001, Wilcoxon rank-sum test) (Table 4).

## DISCUSSION

Our study analyzed 1 year of activities to scale up OTSS in a very low–resource setting and with heterogeneous malaria endemicity.^9^ We evaluated the effects of the OTSS approach on health facility readiness and HCW performance toward meeting clinical and prevention standards during clinical and ANC consultations.

Our findings show that in most of the supervised PHFs, readiness factors, including trained HCWs and availability of malaria case management and MiP guidelines and algorithms, were increasingly available over consecutive rounds of OTSS. For example, using the OTSS data from R1, the NMP conducted additional classroom training, which increased the percentage of HCWs trained from 28% in R1 to 48% in R4. The improvement in this indicator would have been greater if we had also captured onsite training through the readiness checklist. For future supervisions, we suggest that in addition to classroom training, onsite training should also be considered as formal training in the OTSS framework, and the readiness checklist should be updated accordingly.^10^^,^^11^ In addition, almost all supervised PHFs had malaria case management and MiP guidelines and algorithms by R4, which was a significant increase over previous rounds. The Niger NMP and PMI Impact Malaria Project made a special effort to provide PHFs with guidelines and algorithms through any opportunities, such as classroom training, OTSS visits, and other visits and meetings in response to the findings during the first round of OTSS.

Increases in the performance and correct reading of mRDTs in classifying malaria cases, and in managing patients with uncomplicated malaria were documented between R1 and R4. This was similar to results achieved in Nigeria and in the Philippines, where supportive supervision increased health worker performance between baseline and postintervention.^12^^,^^13^ In a study introducing QI methods in the primary health-care system in Bama LGA of Borno State, supportive supervision, also with the aid of a checklist, showed significant improvement in the ability of HCWs to manage children presenting with diarrheal disease.^14^ The analysis of the MiP data set also showed an increase in the performance of HCWs from R1 to R4 in the prevention of MiP and in the malaria case management in pregnant women.

These improvements likely resulted from several important processes instituted by the OTSS approach, including providing instant and individualized feedback and in-service training to the HCWs on steps carried out correctly and incorrectly, which reinforces the knowledge and skills of the HCW. In addition, supervisors worked with facility teams to address their operational bottlenecks, such as lack of supplies and materials. The OTSS approach also provided a steady and sustained emphasis on improving quality over time that reinforced good practices among HCWs.

Similar results have been demonstrated in other countries. In Nepal, a randomized controlled trial of the effects of audit and feedback by district health officers using a structured checklist that focused on prescribing practices in primary health-care facilities resulted in statistically significant differences in adherence to standard treatment schedules.^14^ In an uncontrolled trial, Zeitz et al.^15^ found that supervisor use of a checklist for diarrhea case management during monthly visits to rural health facilities in Nigeria resulted in improvements in history taking, physical examination, disease classification, treatment, and counseling. In Mexico, Kim et al.^16^ found that structured observation and focused performance feedback by supervisors, accompanied by joint identification of opportunities for improvement, increased communication and information sharing to clients by rural doctors.

Factors such as the availability of guidelines and algorithms, and an increased level of training in malaria case management have been shown to benefit HCW performance.^17^ Dedicated supervision ensured by supervisors trained specifically and exclusively for frequent and regular OTSS visits provided recurring opportunities to address issues in service delivery. Whidden et al.^18^ determined that frequent and regular contact may have facilitated the relationship between HCW and supervisor, with both reporting respectful, reliable, and supportive relationships. On the contrary, shortages of mRDTs and/or ACTs and/or insecticide-treated bed nets and/or intermittent preventive treatment during pregnancy may contribute to noncompliance with guidelines even in the face of interventions to improve practices.

Our study had a few important limitations. First, this article presents an analysis of program implementation data, which does not have the level of quality control of the data collected in a controlled study. However, the data collected and analyzed during the implementation of OTSS provided important information and lessons learned for the NMP and other large programs implementing malaria case management interventions in Niger. Furthermore, the use of digital checklists with built-in quality control functions likely mitigated many data quality issues that have been reported previously with use of paper-based data collection. Second, the presence of the supervisor may have caused HCWs to be more attentive and comprehensive in their patient assessments than they would have if not under observation (i.e., the Hawthorne effect^19^). However, this analysis examines trends over successive OTSS visits, when this bias would have been present during each round, thereby reducing any likely effect on the trends documented through this analysis. Last, this analysis assessed HCW performance compared with a standard, but could not assess patients’ clinical outcomes.

It also should be noted that the success of OTSS in Niger and in other countries has depended, to varying degrees, on donor funding, which enables PMI Impact Malaria and other partners to provide technical assistance and logistical support to NMPs. In Niger, where OTSS has only been implemented for a few years in two regions, it is anticipated that ongoing technical and programmatic support for the scale-up of OTSS will be needed in the short to medium terms. The Niger NMP, however, has made an important step toward institutionalization by adopting OTSS as the recommended QI approach for malaria throughout the country. Experience from countries that have implemented OTSS for several years demonstrate that, over time and with appropriate resourcing, a national scale can be achieved, and the need for external support will be reduced as program management is decentralized.^7^^,^^18^^,^^21^

Our analysis demonstrates that significant improvements were identified in PHF readiness and HCW performance in meeting clinical and prevention standards for malaria management in outpatient and antenatal consultations between the first round in July 2020 and the fourth round in August 2021. This assessment provides further evidence that the OTSS approach to QI can achieve rapid improvements in the readiness of health facilities and the competency of health workers managing outpatients and ANC clients. Expansion of OTSS to other areas of Niger are likely to yield similar benefits. Efforts to expand competency-based supervision to the community level are underway. If successful, the improvement of quality throughout the continuum of care is likely to improve outcomes of those seeking care for malaria.

## Supplemental Materials

10.4269/ajtmh.23-0359 Supplemental Materials
