# Supplementary material for: How Outreach Training and Supportive Supervision (OTSS) Affect Health Facility Readiness and Health-Care Worker Competency to Prevent and Treat Malaria in Niger: A Secondary Analysis of OTSS Data
Source: Am J Trop Med Hyg. 2024 Feb 6;110(3 Suppl):50–5. doi: 10.4269/ajtmh.23-0359 (PMC10919237; doi:10.4269/ajtmh.23-0359)
Supplement: Supplemental Materials [file tpmd230359.SD1.pdf]

Tab / Header / Question - English	Score Numerator	Score Denominator	Composite Indicator
**Information**			
Does facility offer ANC services?			
Does facility distribute LLINs to children under 5 years of age?			
Does facility offer laboratory services?			
Does facility offer diazepam?			
Does facility offer blood transfusion?			
Is there at least 1 CHW attached to this facility?			
Does facility offer case management of severe malaria services?			
END OF TAB			
**Availability of medications**	**28.0**	**28.0**	**1**
Are these medications available today?			
Paracetamol	1.0	1.0	1
Sulfadoxine-Pyrimethamine	2.0	2.0	1
ACTs for children under 5 years	2.0	2.0	1
ACTs for children 5-7 years	2.0	2.0	1
ACTs for children 7-13 years	2.0	2.0	1
ACTs for children over 13 and adults	2.0	2.0	1
Injectable Quinine	2.0	2.0	1
Compressed Quinine	2.0	2.0	1
Injectable Artesunate and/or IM Injectable Artemether	2.0	2.0	1
Artesunate Rectocaps	2.0	2.0	1
Iron Tablets	1.0	1.0	1
0.4 mg Folic Acid	1.0	1.0	1
Diazepam	1.0	1.0	1
5% Glucose	2.0	2.0	1
10% Glucose	1.0	1.0	1
30% Glucose	1.0	1.0	1
Lactate Ringer	1.0	1.0	1
Saline Solution	1.0	1.0	1
END OF TAB			
**Availability of commodities**	**29.0**	**29.0**	**2**
Are these commodities available today?			
Oxygen	3.0	3.0	2
Drinking water at the sie of ANC	2.0	2.0	2
Disposable glasses available (or consistent cleaning) for the DOT strategy of the SP during ANC	1.0	1.0	2
Examination gloves	2.0	2.0	2
Soap or hand sanitizer gel	1.0	1.0	2
Sharps containers	3.0	3.0	2
Themometer	2.0	2.0	2
Personal scale	2.0	2.0	2

Syringes	2.0	2.0	2
Alcohol	1.0	1.0	2
Cotton	1.0	1.0	2
Malaria RDTs	3.0	3.0	2
LLINs	3.0	3.0	2
Reagents for determining blood groups	2.0	2.0	2
Blood for transfusion	1.0	1.0	2
END OF TAB			
**Availability of documentation**	**11.0**	**11.0**	**3**
Are these documents available today?			
Records of curative consultations	2.0	2.0	3
Records of prenatal consultations	2.0	2.0	3
Records of the distribution of LLINs to children under 5 years of age	1.0	1.0	3
Records of the distribution of LLINs to pregnant women	1.0	1.0	3
HMIS reports	2.0	2.0	3
Monthly CHW reports	2.0	2.0	3
Manual for filling out the data collection media	1.0	1.0	3
END OF TAB			
**Availability of materials**	**9.0**	**9.0**	**4**
Are these materials (guides, job aides, etc) available today?			
Malaria and pregnancy guide or tools/support on ANC	1.0	1.0	4
ANC guidelines	1.0	1.0	4
National guidelines for the management of malaria	2.0	2.0	4
Management of uncomplicated malaria fact sheet	1.0	1.0	4
Management of severe malaria fact sheet	1.0	1.0	4
Management of malaria in pregnancy fact sheet	1.0	1.0	4
Preparation of artesunate injection PEC fact sheet	1.0	1.0	4
LLIN distribution guidelines	1.0	1.0	4
END OF TAB			
**Availability of trained human resources**	**3.0**	**3.0**	**5**
Number of health workers by category (treating malaria): To be broken down by gender			
Physicians - Male			5
Physicians - Female			5
Midwives - Male			5
Midwives - Female			5
Nurses - Male			5
Nurses - Female			5
Pharmacists/PGP - Male			5
Pharmacists/PGP - Female			5
Does the health facility have at least 50% of staff trained to manage malaria cases using the latest national guidelines?	3.0	3.0	5
END OF TAB			
**Availability of quality data**	**7.0**	**7.0**	**6**
**Number of pregnant women received in ANC 1**	**1.0**	**1.0**	**6.1**

Amount reported (R)			6.1
Amount found in the register (F)			6.1
Difference (F-R) <= 0?	1.0	1.0	6.1
**Number of pregnant women who received IPT 3**	**1.0**	**1.0**	**6.2**
Amount reported (R)			6.2
Amount found in the register (F)			6.2
Difference (F-R) <= 0?	1.0	1.0	6.2
**Number of suspected malaria cases registered in the health facility**	**1.0**	**1.0**	**6.3**
Amount reported (R)			6.3
Amount found in the register (F)			6.3
Difference (F-R) <= 0?	1.0	1.0	6.3
**Number of suspected malaria cases tested by RDT or thick smear test**	**1.0**	**1.0**	**6.4**
Amount reported (R)			6.4
Amount found in the register (F)			6.4
Difference (F-R) <= 0?	1.0	1.0	6.4
**Number of uncomplicated malaria cases tested by RDT or thick smear test**	**1.0**	**1.0**	**6.5**
Amount reported (R)			6.5
Amount found in the register (F)			6.5
Difference (F-R) <= 10?	1.0	1.0	6.5
**Number of confirmed uncomplicated malaria cases treated with ACTs**	**1.0**	**1.0**	**6.6**
Amount reported (R)			6.6
Amount found in the register (F)			6.6
Difference (F-R) <= 0?	1.0	1.0	6.6
**Number of ACT treatments consumed (taken from the health facility's pharmacy)**	**1.0**	**1.0**	**6.7**
Amount reported (R)			6.7
Amount found in the register (F)			6.7
Difference (F-R) <= 0?	1.0	1.0	6.7
END OF TAB			

**Overall QA Score**	**0**
**Availability of medications**	**1**
**Availability of commodities**	**2**
**Availability of documentation**	**3**
**Availability of materials**	**4**
**Availability of trained human resources**	**5**
**Availability of quality data**	**6**
Number of pregnant women received in ANC 1	6.1
Number of pregnant women who received IPT 3	6.2
Number of suspected malaria cases registered in the health facility	6.3
Number of suspected malaria cases tested by RDT or thick smear test	6.4
Number of uncomplicated malaria cases tested by RDT or thick smear test	6.5
Number of confirmed uncomplicated malaria cases treated with ACTs	6.6

Number of ACT treatments consumed (taken from the health facility's pharmacy)	6.7

Mandatory	Tab / Header / Question - English	Tab / Header / Question - French (original)	Score Numerator	Score Denominator	Composite Indicator
	**Clinical**		**89.0**	**89.0**	**0**
	**Reception**		**4.0**	**4.0**	**1**
*	Did the provider greet the patient at the entrance of the consultation office?		1.0	1.0	1
*	Did the provider invite the patient to sit down?		1.0	1.0	1
*	Did the provider introduce themself to the patient?		1.0	1.0	1
*	Did the provider treat the patient with kindness and respect (Listening with attention, showing interest to the woman as a person) throughout the visit?		1.0	1.0	1
	**Collecting historical patient information**		**15.0**	**15.0**	**2**
*	Did the provider ask the patient's age?		2.0	2.0	2
*	Is the patient over 12 years old?				2
*	Is the patient a woman of childbearing age?				2
*	Did the provider ask where the patient lived?		1.0	1.0	2
*	Did the provider look for a sign of fever or ask about a history of fever over the two previous days?		2.0	2.0	2
*	Did the health provider ask about the symptoms of uncomplicated malaria (fever, chills, sweating, headache, aches, nausea, lack of appetite, etc.)?		2.0	2.0	2
*	Did the health provider ask questions looking for signs of severe malaria in the patient (convulsions, agitation, confusion, prostration, difficulty breathing, spontaneous bleeding, dark urine, etc.)?		2.0	2.0	2
*	If a woman of childbearing age, has the health provider asked about pregnancy status or DDR?		2.0	2.0	2
*	Did the provider ask if the patient has taken any malaria treatment in the last two weeks?		2.0	2.0	2
*	Did the health provider ask about any drugs already taken by the patient?		2.0	2.0	2
	**Diagnosis**		**22.0**	**22.0**	**3**
*	Did the provider take the temperature?		2.0	2.0	3
*	Did the provider measure the patient’s weight?		2.0	2.0	3
*	Did the provider take the patient’s blood pressure?		2.0	2.0	3
*	Did the provider take the patient’s pulse?		2.0	2.0	3
*	Did the provider take the patient’s respiratory rate?		2.0	2.0	3
*	Did the health provider look for signs of anemia in the patient (conjunctivae, palms)?		3.0	3.0	3
*	Did the health provider look for signs of other serious illnesses (eyes, neck, etc.)?		3.0	3.0	3
*	Did the provider perform a complete physical examination of the patient (eyes, conjunctivae, fundus, palms, ears, throat, neck, heart, lungs, abdomen, and reflex)?		3.0	3.0	3
*	Did the provider request a biological test to confirm malaria?		3.0	3.0	3
	**Classification of Malaria**		**5.0**	**5.0**	**4**

*	What was the result of the test?			4
*	What type of malaria was categorized by the health worker?			4
*	Does the supervisor agree with the provider's malaria classification?	3.0	3.0	4
*	Did the provider explain the meaning of the result(positive or negative) of the test to the patient and/or family and detail the appropriate course of action?	2.0	2.0	4
	**Adherence to negative test result**	**2.0**	**2.0**	**5.1**
*	Has the provider refrained from prescribing or administering an antimalarial drug to the patient based on the NEGATIVE result of the malaria laboratory test (RDT or GE)?	2.0	2.0	5.1
	**Treatment for uncomplicated malaria**	**18.0**	**18.0**	**5.2**
*	Is the patient pregnant?			5.2
*	Did the provider use a recommended ACT to treat uncomplicated malaria in the patient as directed by national guidelines? (non-pregnant)	3.0	3.0	5.2
*	If the patient is pregnant, what is the gestational age?			5.2
*	Did the agent use an ACT recommended to treat uncomplicated malaria in patients including pregnant women in the 2nd and 3rd trimester of pregnancy as directed?	2.0	2.0	5.2
*	If the patient is a pregnant woman in the first trimester of pregnancy, has the Provider used quinine tablets to treat uncomplicated malaria according to national guidelines?	3.0	3.0	5.2
*	Did the provider use the correct dosage to treat uncomplicated malaria?	3.0	3.0	5.2
*	Is the dosage and duration of treatment explained to the patient?	3.0	3.0	5.2
*	Did the provider verify that the patient understood the explanations given?	1.0	1.0	5.2
*	Did the provider inform the patient of the potential undesirable effects of the drug used?	1.0	1.0	5.2
*	Did the provider invite the patient back if he/she has complications?	1.0	1.0	5.2
*	Did the provider give the patient a follow-up appointment?	1.0	1.0	5.2
	**Pre-transfer treatment for severe malaria**	**17.0**	**17.0**	**5.3**
*	Did the provider administer a pre-transfer treatment to the patient?	3.0	3.0	5.3
*	Which pre-transfer treatment did the Provider administer to the patient?			5.3
*	If artesunate was used for the pre-transfer treatment, did the provider calculate the correct amount based on the patient's weight?	3.0	3.0	5.3
*	i- Removed and injected the entire contents (1 ml) of the sodium bicarbonate ampoule into the artesunate powder vial	0.75	0.75	5.3
*	ii- shook gently until dissolved and a clear reconstituted solution was obtained	0.75	0.75	5.3

*	iii- removed and injected the required volume of 5% saline or dextrose solution (depending on IV or IM) into the reconstituted artesunate solution	0.7 5	0.75	5.3
*	iv- removed the dose (in ml) required for IV or IM administration from the vial(s) prepared beforehand and injected the solution	0.7 5	0.75	5.3
*	Did the provider administer a weight-appropriate dose of artesunate based on IV or IM route?	3.0	3.0	5.3
*	If artemether injection is used for pre-transfer treatment, is the dosage as directed?	3.0	3.0	5.3
*	Did the Provider provide a reference note to the patient's parents?	2.0	2.0	5.3
	**Conclusion of visit**	**4.0**	**4.0**	**6**
*	Did the provider record all the information collected from the patient in their register?	2.0	2.0	6
*	Did the provider provide to the patient the opportunity to ask question?	1.0	1.0	5.2
*	Did the provider give the patient and his/her family appropriate advice on malaria (causes, risks, prevention)?	1.0	1.0	5.2
	END OF TAB			
	**Composite Scores**			
	**Overall QA Score**			**0**
	**Reception**			**1**
	**Collecting historical patient information**			**2**
	**Diagnosis**			**3**
	**Classification of malaria**			**4**
	**Adherence to test result**			**5**
	Adherence to negative test result			5.1
	Treatment of Uncomplicated Malaria			5.2
	Pre-transfer treatment for severe malaria			5.3
	**Conclusion of visit**			**6**

Tab / Header / Question - English	Score Numerator	Score Denominator	Composite Indicator
**Observation**	**104.0**	**104.0**	**0**
**Reception**	**4.0**	**4.0**	**1**
Did the provider greet the woman at the entrance of the consultation office?	1.0	1.0	1
Did the provider invite the woman to sit down?	1.0	1.0	1
Did the provider introduce themself to the woman?	1.0	1.0	1
Did the provider treat the woman with kindness and respect (Listening with attention, showing interet to the woman as a person) throughout the visit?	1.0	1.0	1
**Collecting historical patient information**	**13.0**	**13.0**	**2**
Did the provider ask the woman's age?	1.0	1.0	2
Did the provider ask where the woman lived?	1.0	1.0	2
Did the provider research the gestational period, date of last menstrual period, ultrasound, fundal height, ANC booklet?	3.0	3.0	2
Did the provider ask if the patient has taken any malaria treatment in the last two weeks?	2.0	2.0	2
Did the provider ask about any drugs already taken by the woman?	2.0	2.0	2
Did the provider research the gestational period, date of last menstrual period, ultrasound, fundal height, ANC booklet?	2.0	2.0	2
Did the provider ask or investigate whether the patient was at her first CPN?	2.0	2.0	2
**MIP Case Management**	**49.0**	**49.0**	**3**
**Malaria diagnosis**	**28.0**	**28.0**	**3.1**
Did the provider look for a sign of fever or ask about a history of fever during the previous 2 days?	2.0	2.0	3.1
Did patient have fever, signs of fever, or history of fever?			3.1
Did the provider ask about the symptoms of uncomplicated malaria (fever, chills, sweating, headache, aches, nausea, lack of appetite, etc.)?	2.0	2.0	3.1
Did the provider ask questions looking for signs of severe malaria (convulsions, agitation, confusion, prostration, etc.)?	2.0	2.0	3.1
Did the provider take the temperature?	2.0	2.0	3.1
Did the provider measure the patient’s weight?	2.0	2.0	3.1
Did the provider take the patient's blood pressure?	2.0	2.0	3.1
Did the provider take the patient's pulse?	2.0	2.0	3.1
Did the provider take the patient's respiratory rate?	2.0	2.0	3.1
Did the provider check for fetal heart sounds (2nd and 3rd trimester of pregnancy)?	3.0	3.0	3.1
Did the provider look for signs of anemia in the woman (pallor of the conjunctivae/palms of hands)?	3.0	3.0	3.1
Did the provider look for signs of other serious illnesses (Urinary tract infections, hypertensive disorders etc.)?	3.0	3.0	3.1
Did the provider request a biological test to confirm malaria (RDT/GE)?	3.0	3.0	3.1
What was the result of the biological examination?			3.1
If the result of the malaria test was positive, did the provider explain the result to the patient?	3.0	3.0	3.1
Did the provider categorize the severity of malaria according to national guidelines?	3.0	3.0	3.1

What type of malaria was categorized by the health worker?			3.1
**Antimalarial treatment**	**21.0**	**21.0**	**3.2**
If it is uncomplicated malaria, does the antimalarial drug used to treat the patient comply with the guidelines?	3.0	3.0	3.2
Did the worker use the correct dosage to treat uncomplicated malaria in pregnant women?	3.0	3.0	3.2
Did the provider explain the dosage and duration of the drug to the patient?	2.0	2.0	3.2
Did the provider verify that the patient understood the explanations given?	1.0	1.0	3.2
Did the provider inform the patient of the potential undesirable effects of the drug used?	1.0	1.0	3.2
Did the provider invite the patient to come back if she has complications?	1.0	1.0	3.2
Did the provider give the patient a follow-up appointment?	1.0	1.0	3.2
Did the provider enter information on the treatment into the register?	1.0	1.0	3.2
If it is severe malaria, did the provider administer any pre-transfer treatment?	3.0	3.0	3.2
Does the drug used for pre-transfer treatment of severe malaria in pregnant women comply with the guidelines?	2.0	2.0	3.2
Did the provider record all the information collected from the patient in the register?	1.0	1.0	3.2
Has the patient and/or her family received appropriate malaria counseling?	1.0	1.0	3.2
Did the provider verify that the patient understood the advice?	1.0	1.0	3.2
**MIP Prevention**	**36.0**	**36.0**	**4**
**Dispensing IPTp**	**26.0**	**26.0**	**4.1**
Did the provider ask or investigate whether the patient had taken SP in IPT during the previous four weeks?	2.0	2.0	4.1
Did the provider check (mother-child record) whether the patient was on co-trimoxazole for HIV chemoprevention?	2.0	2.0	4.1
Did the provider communicate with the patient about the consequences of malaria in pregnancy?	1.0	1.0	4.1
Did the provider make the patient aware of the benefits of preventing malaria with SP?	1.0	1.0	4.1
Did the provider educate the patient about the need to take at least three doses of SP before giving birth?	1.0	1.0	4.1
Did the provider verify whether the patient is eligible for IPT during this ANC?	3.0	3.0	4.1
Is the patient eligible according to the above criteria?			4.1
If not eligible, specify why			4.1
Did the provider inform the patient about the potential side effects of SP?	1.0	1.0	4.1
Did the provider give three SP tablets to the patient eligible for IPT?	3.0	3.0	4.1
Did the provider ask the patient to drink water to swallow the three SP tablets on the spot?	3.0	3.0	4.1
Did the provider observe the patient taking the three SP tablets?	3.0	3.0	4.1
Did the provider encourage the patient to return each month for IPT up until delivery?	2.0	2.0	4.1

Did the provider ask the patient to return to see them if there are any serious skin and/or digestive symptoms as a result of taking the SP?	1.0	1.0	4.1
Did the provider inform the patient of the date of the next appointment?	2.0	2.0	4.1
Did the provider record the dose of SP in the mother-child record and ANC registry in compliance with the national guidelines?	1.0	1.0	4.1
**LLIN Distribution**	**7.0**	**7.0**	**4.2**
Did the provider explain the benefits of sleeping under an LLIN every night to prevent malaria?	1.0	1.0	4.2
Did the pregnant woman receive an LLIN for this pregnancy during the previous visits?			4.2
Did the provider give the patient an LLIN if she had not yet received it during this pregnancy?	3.0	3.0	4.2
Did the provider explain to the patient how to use the LLIN?	1.0	1.0	4.2
Did the provider advise the patient to sleep under LLINs during pregnancy and after delivery?	1.0	1.0	4.2
Did the provider record the provision of the LLIN to the patient in the mother-child record and in the ANC register?	1.0	1.0	4.2
**Dispensing anti-anemia drugs**	**3.0**	**3.0**	**4.3**
Did the provider give the patient iron tablets after the consultation?	1.0	1.0	4.3
Did the provider give the patient 0.4 mg folic acid tablets after the consultation?	1.0	1.0	4.3
Did the provider check that the patient understood how to take the iron and folic acid tablets?	1.0	1.0	4.3
**Conclusion of visit**	**2.0**	**2.0**	**5**
Did the health worker provide the opportunity to the woman to ask questions?	1.0	1.0	5
Did the provider provide the patient and her family appropriate advice on malaria (causes, risks, prevention)?	1.0	1.0	5
END OF TAB			
**Composite Scores**			
**Overall QA Score**			**0**
**Reception**			**1**
**Collecting historical patient information**			**2**
**MIP Case Management**			**3**
Malaria diagnosis			3.1
Antimalarial treatment			3.2
**MIP Prevention**			**4**
Dispensing IPTp			4.1
LLIN Distribution			4.2
Dispensing anti-anemia drugs			4.3
**Conclusion of visit**			**5**

Figure S2: Map of Dosso and Tahoua regions showing targeted PHFs

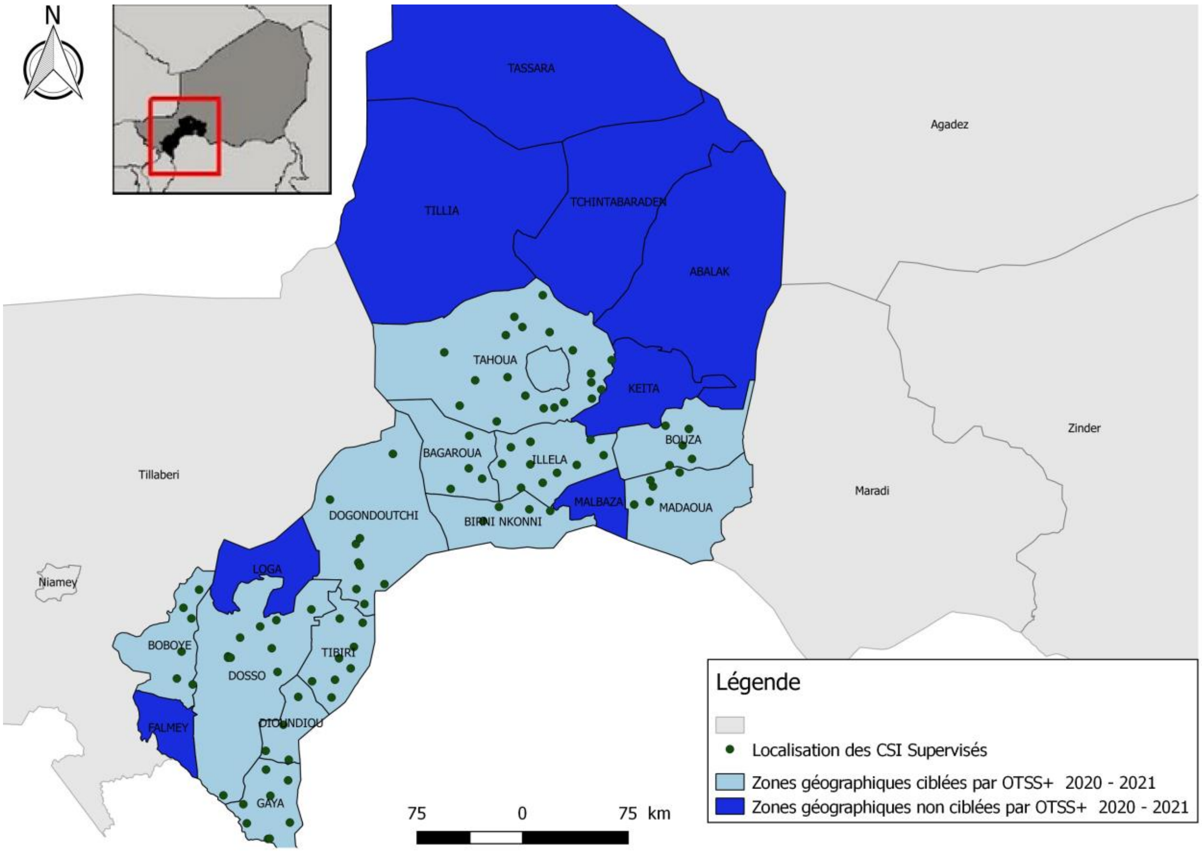

SUPPLEMENTAL MATERIAL

Components of indicators used in the analysis.

Indicators	Checklist	Checklist Components
The availability of trained staff, malaria rapid diagnostic test (mRDT)	Facility Readiness	Does the health facility have at least 50% of staff trained to manage malaria cases using the latest national guidelines?
The availability of malaria rapid diagnostic test (mRDT	Facility Readiness	Are malaria rapid diagnostic test (mRDTs) available today?
The availability of at least one formulation of ACT	Facility Readiness	Are these medications available today?
		ACTs for children under 5 years
		ACTs for children 5-7 years
		ACTs for children 7-13 years
		ACTs for children over 13 and adults
The availability of case management and MiP guidelines/algorithms	Facility Readiness	Are these materials (guides, job aides, etc) available today?
		Malaria and pregnancy guide or tools/support on ANC
		ANC guidelines
		National guidelines for the management of malaria
		Management of uncomplicated malaria fact sheet
		Management of severe malaria fact sheet
		Management of malaria in pregnancy fact sheet
		Preparation of artesunate injection PEC fact sheet
		LLIN distribution guidelines
HCW competency in using and reading mRDTs	mRDT Observation	RDT Preparation
		Did the provider check the expiration date?
		Did the provider label the cassette with client’s name/ID number?
		Client Preparation
		Was the client identified and their identification information recorded in register?
		Did the provider explain the procedure to client?
		Clean gloves are worn?
		The puncture point is cleaned with alcohol and allowed to air dry
		Blood collection
		Did the provider collect an adequate volume of blood?
		Did the provider deposit blood in correct well of RDT device?
		RDT Procedure
		Did the provider deposit buffer to correct well of RDT device?

Indicators	Checklist	Checklist Components
		Did the provider dispense the appropriate number of drops of butter into well?
		What was the result?
		If negative, did the provider wait for correct incubation time according to manufacturer's instruction?
		Is the control line visible?
		Did the agent conclude that the test was invalid if no band appeared in the control line C?
		Did the agent read the results correctly (the supervisor checks the results)
		Did the provider read test results correctly in register?
		Waste Management
		Were used tests, transfer devices and other blood-contaminated material disposed of appropriately?
		Was the used lancet disposed in sharps container?
HCW adherence to negative mRDT results	Case management	Has the provider refrained from prescribing or administering an antimalarial drug to the patient based on the NEGATIVE result of the malaria laboratory test (RDT or GE)?
HCW competency of classifying malaria cases as uncomplicated or severe	Case management	What was the result of the test?
		What type of malaria was categorized by the health worker?
		Does the supervisor agree with the provider's malaria classification?
		Did the provider explain the meaning of the result (positive or negative) of the test to the patient and/or family and detail the appropriate course of action?
HCW competency in managing patients with uncomplicated malaria.	Case management	Reception
		Did the provider greet the patient at the entrance of the consultation office?
		Did the provider invite the patient to sit down?
		Did the provider introduce themself to the patient?
		Did the provider treat the patient with kindness and respect (Listening with attention, showing interest to the woman as a person) throughout the visit?
		Collecting Historical Patient Information
		Did the provider ask the patient's age?
		Is the patient over 12 years old?
		Is the patient a woman of childbearing age?
		Did the provider ask where the patient lived?
		Did the provider look for a sign of fever or ask about a history of fever over the two previous days?
		Did the health provider ask about the symptoms of uncomplicated malaria (fever,

Indicators	Checklist	Checklist Components
		chills, sweating, headache, aches, nausea, lack of appetite, etc.)?
		Did the health provider ask questions looking for signs of severe malaria in the patient (convulsions, agitation, confusion, prostration, difficulty breathing, spontaneous bleeding, dark urine, etc.)?
		If a woman of childbearing age, has the health provider asked about pregnancy status or DDR?
		Did the provider ask if the patient has taken any malaria treatment in the last two weeks?
		Did the health provider ask about any drugs already taken by the patient?
		Diagnosis
		Did the provider take the temperature?
		Did the provider measure the patient's weight?
		Did the provider take the patient's blood pressure?
		Did the provider take the patient's pulse?
		Did the provider take the patient's respiratory rate?
		Did the health provider look for signs of anemia in the patient (conjunctivae, palms)?
		Did the health provider look for signs of other serious illnesses (eyes, neck, etc.)?
		Did the provider perform a complete physical examination of the patient (eyes, conjunctivae, fundus, palms, ears, throat, neck, heart, lungs, abdomen, and reflex)?
		Did the provider request a biological test to confirm malaria?
		Classification of Malaria
		What was the result of the test?
		What type of malaria was categorized by the health worker?
		Does the supervisor agree with the provider's malaria classification?
		Did the provider explain the meaning of the result (positive or negative) of the test to the patient and/or family and detail the appropriate course of action?
		Treatment of Uncomplicated Malaria
		Did the provider use a recommended ACT to treat uncomplicated malaria in the patient as directed by national guidelines?
		Is the patient pregnant?
		If the patient is pregnant, what is the gestational age?
		Did the agent use an ACT recommended to treat uncomplicated malaria in patients including pregnant women in the 2nd and 3rd trimester of pregnancy as directed?
		If the patient is a pregnant woman in the first trimester of pregnancy, has the Provider

Indicators	Checklist	Checklist Components
		used quinine tablets to treat uncomplicated malaria according to national guidelines?
		Did the provider use the correct dosage to treat uncomplicated malaria?
		Is the dosage and duration of treatment explained to the patient?
		Did the provider verify that the patient understood the explanations given?
		Did the provider inform the patient of the potential undesirable effects of the drug used?
		Did the provider invite the patient back if he/she has complications?
		Did the provider give the patient a follow-up appointment?
		Pre-transfer treatment for severe malaria
		Did the provider administer a pre-transfer treatment to the patient?
		Which pre-transfer treatment did the Provider administer to the patient?
		If artesunate was used for the pre-transfer treatment, did the provider calculate the correct amount based on the patient's weight?
		Check if the agent follows the procedure for the preparation of injectable artesunate:<br>i- Removed and injected the entire contents (1 ml) of the sodium bicarbonate ampoule into the artesunate powder vial.<br>ii- shook gently until dissolved and a clear reconstituted solution was obtained.<br>iii- removed and injected the required volume of 5% saline or dextrose solution (depending on IV or IM) into the reconstituted artesunate solution.<br>iv- removed the dose (in ml) required for IV or IM administration from the vial(s) prepared beforehand and injected the solution
		Did the provider administer a weight-appropriate dose of artesunate based on IV or IM route?
		If artemether injection is used for pre-transfer treatment, is the dosage as directed?
		Did the Provider provide a reference note to the patient's parents?
		Conclusion of the visit
		Did the provider record all the information collected from the patient in their register?
		Did the provider provide to the patient the opportunity to ask question?
		Did the provider give the patient and his/her family appropriate advice on malaria (causes, risks, prevention)?
HCW competency in the prevention of malaria in pregnancy	Malaria in Pregnancy	Reception
		Did the provider greet the woman at the entrance of the consultation office?
		Did the provider invite the woman to sit down?
		Did the provider introduce themself to the woman?

Indicators	Checklist	Checklist Components
		Did the provider treat the woman with kindness and respect (Listening with attention, showing interest to the woman as a person) throughout the visit?
		Collecting historical information
		Did the provider ask the woman's age?
		Did the provider ask where the woman lived?
		Did the provider research the gestational period, date of last menstrual period, ultrasound, fundal height, ANC booklet?
		Did the provider ask if the patient has taken any malaria treatment in the last two weeks?
		Did the provider ask about any drugs already taken by the woman?
		Did the provider research the gestational period, date of last menstrual period, ultrasound, fundal height, ANC booklet?
		Did the provider ask or investigate whether the patient was at her first CPN?
		Dispensing IPTp
		Did the provider ask or investigate whether the patient had taken SP in IPT during the previous four weeks?
		Did the provider check (mother-child record) whether the patient was on co-trimoxazole for HIV chemoprevention?
		Did the provider communicate with the patient about the consequences of malaria in pregnancy?
		Did the provider make the patient aware of the benefits of preventing malaria with SP?
		Did the provider educate the patient about the need to take at least three doses of SP before giving birth?
		Did the provider verify whether the patient is eligible for IPT during this ANC?
		Is the patient eligible according to the above criteria?
		If not eligible, specify why
		Did the provider inform the patient about the potential side effects of SP?
		Did the provider give three SP tablets to the patient eligible for IPT?
		Did the provider ask the patient to drink water to swallow the three SP tablets on the spot?
		Did the provider observe the patient taking the three SP tablets?
		Did the provider encourage the patient to return each month for IPT up until delivery?
		Did the provider ask the patient to return to see them if there are any serious skin and/or digestive symptoms as a result of taking the SP?

Indicators	Checklist	Checklist Components
		Did the provider inform the patient of the date of the next appointment?
		Did the provider record the dose of SP in the mother-child record and ANC registry in compliance with the national guidelines?
		ITN Distribution
		Did the provider explain the benefits of sleeping under an LLIN every night to prevent malaria?
		Did the pregnant woman receive an LLIN for this pregnancy during the previous visits?
		Did the provider give the patient an LLIN if she had not yet received it during this pregnancy?
		Did the provider explain to the patient how to use the LLIN?
		Did the provider advise the patient to sleep under LLINs during pregnancy and after delivery?
		Did the provider record the provision of the LLIN to the patient in the mother-child record and in the ANC register?
		Dispensing antianemia drugs
		Did the provider give the patient iron tablets after the consultation?
		Did the provider give the patient 0.4 mg folic acid tablets after the consultation?
		Did the provider check that the patient understood how to take the iron and folic acid tablets?
HCW competency in managing pregnant women with malaria.	Malaria in Pregnancy	Reception
		Did the provider greet the woman at the entrance of the consultation office?
		Did the provider invite the woman to sit down?
		Did the provider introduce themself to the woman?
		Did the provider treat the woman with kindness and respect (Listening with attention, showing interest to the woman as a person) throughout the visit?
		Collecting Historical Patient Information
		Did the provider ask the woman's age?
		Did the provider ask where the woman lived?
		Did the provider research the gestational period, date of last menstrual period, ultrasound, fundal height, ANC booklet?
		Did the provider ask if the patient has taken any malaria treatment in the last two weeks?
		Did the provider ask about any drugs already taken by the woman?

Indicators	Checklist	Checklist Components
		Did the provider research the gestational period, date of last menstrual period, ultrasound, fundal height, ANC booklet?
		Did the provider ask or investigate whether the patient was at her first CPN?
		Malaria Diagnosis
		Did the provider look for a sign of fever or ask about a history of fever during the previous 2 days?
		Did patient have fever, signs of fever, or history of fever?
		Did the provider ask about the symptoms of uncomplicated malaria (fever, chills, sweating, headache, aches, nausea, lack of appetite, etc.)?
		Did the provider ask questions looking for signs of severe malaria (convulsions, agitation, confusion, prostration, etc.)?
		Did the provider take the temperature?
		Did the provider measure the patient’s weight?
		Did the provider take the patient's blood pressure?
		Did the provider take the patient's pulse?
		Did the provider take the patient's respiratory rate?
		Did the provider check for fetal heart sounds (2nd and 3rd trimester of pregnancy)?
		Did the provider look for signs of anemia in the woman (pallor of the conjunctivae/palms of hands)?
		Did the provider look for signs of other serious illnesses (Urinary tract infections, hypertensive disorders etc.)?
		Did the provider request a biological test to confirm malaria (RDT/GE)?
		What was the result of the biological examination?
		If the result of the malaria test was positive, did the provider explain the result to the patient?
		Did the provider categorize the severity of malaria according to national guidelines?
		What type of malaria was categorized by the health worker?
		Antimalarial treatment
		If it is uncomplicated malaria, does the antimalarial drug used to treat the patient comply with the guidelines?
		Did the worker use the correct dosage to treat uncomplicated malaria in pregnant women?
		Did the provider explain the dosage and duration of the drug to the patient?
		Did the provider verify that the patient understood the explanations given?

Indicators	Checklist	Checklist Components
		Did the provider inform the patient of the potential undesirable effects of the drug used?
		Did the provider invite the patient to come back if she has complications?
		Did the provider give the patient a follow-up appointment?
		Did the provider enter information on the treatment into the register?
		If it is severe malaria, did the provider administer any pre-transfer treatment?
		Does the drug used for pre-transfer treatment of severe malaria in pregnant women comply with the guidelines?
		Did the provider record all the information collected from the patient in the register?
		Has the patient and/or her family received appropriate malaria counseling?
		Did the provider verify that the patient understood the advice?
